# The Characterization of Laser-Induced Particles Generated from Aluminum Alloy in High Power Laser Facility

**DOI:** 10.3390/ma16237415

**Published:** 2023-11-29

**Authors:** Xinxiang Miao, Guorui Zhou, Qihua Zhu, Xiaodong Jiang, Yong Jiang, Caizhen Yao, Yilan Jiang, Longfei Niu, Siheng Xiang, Jiaxuan Chen

**Affiliations:** 1 Laser Fusion Research Center, China Academy of Engineering Physics, Mianyang 621900, China; miaoxx@caep.cn (X.M.); zhougr@caep.cn (G.Z.); jiangxdong@163.com (X.J.); yaocaizhen2008@126.com (C.Y.); jiangyilan1023@163.com (Y.J.); niulf@caep.cn (L.N.); xiangsiheng1230@163.com (S.X.); 2 School of Mathematics and Physics, Southwest University of Science and Technology, Mianyang 621010, China; 3 Center for Precision Engineering, Harbin Institute of Technology, Harbin 150001, China; chenjiaxuan@hit.edu.cn

**Keywords:** contamination particles, high energy laser system, thermodynamic ablation, laser-induced damage, clean control

## Abstract

Aerosol particle contamination in high-power laser facilities has become a major cause of internal optical component damage resistance and service life reduction. In general, contaminating particles primarily originate from stray light; therefore, it is crucial to investigate the mechanism and dynamics of the dynamic contaminating particle generation to control the cleanliness level. In this study, corresponding research was conducted on experiments and theory. We investigated the particle generation and surface composition modification under the action of a laser. We employed various surface analytical methods to identify the possible variations in the aluminum alloy surface during laser irradiations. A theoretical model for particle ejection from aluminum alloy surfaces was established by taking the adhesion force and laser cleaning force (due to thermal expansion) into account. The results show that the threshold energies for contamination particle generation and damage are around 0.1 and 0.2 J/cm^2^, respectively. Subsurface impurities are the primary source of particles, and particle adhesion density is related to surface roughness. Pollution particle generation and splashing processes include temperature increases, phase changes, impact diffusion, and adhesion. The results provide a reference for the normal operation of high-energy laser systems. The results also suggest that the laser irradiation pretreatment of aluminum alloy surfaces is essential to improve the cleanliness level.

## 1. Introduction

Recently, research on laser inertial confinement fusion (ICF) has intensified with the successful ignition of the National Ignition Facility (NIF), built at the Lawrence Livermore National Laboratory (LLNL) in the USA. To achieve fusion conditions, the output energy of a high-power laser facility must be increased to 2.05 MJ [1,2], and its corresponding triple-frequency energy must be up to 8 J/cm^2^. 

In the ICF high-energy laser system, an ultraviolet laser is ultimately used, which can easily cause damage to components, such as fused quartz, and has become one of the bottlenecks limiting system energy improvement [3,4,5]. Despite continued improvements in damage performance, the surface damage threshold of the components is still far below the intrinsic bulk limit of the material [6]. Recent work shows there are many sources of laser-induced damage to fused silica components, such as scratches [7], subsurface defects [8], inclusions [9], damage precursors [10], and surface contamination [11,12], which cause the most significant reduction in the laser-induced damage threshold (LIDT) of fused silica [13]. These near-surface defects (damage precursors) can absorb the subband-ccgap light, leading to damage to optical materials. Thus, the fused silica was treated with an acid etch to improve the resistance to laser damage caused by the damage precursors during polishing. This technology was named the Advanced Mitigation Process (AMP), which used an optimized hydrofluoric (HF) acid or a buffered oxide etch (BOE) with ultrasonic and megasonic assistance to remove both the polishing layer and fracture-induced electronic defect layer. This process can enhance the surface damage threshold (351 nm) up to ~40–50 J/cm^2^ [6]. However, there are no effective methods for surface contamination control.

The optical properties of surface contamination determine the nature of the optical damage. Metallic particles with high intrinsic optical absorption often absorb laser energy, leading to a formation of confined plasma and subsequent etching of the fused surface [14]. Particles such as Fe and Al_2_O_3_ on the surface of fused silica components can degrade the LIDT of these components by more than 50% [15,16,17]. In addition, previous studies have revealed that aluminum particles on the surface of fused silica components not only cause damage to the optical surfaces but also modulate the subsequent laser, which causes the particles to melt and vaporize under laser irradiation with a fluence of 3 J/cm^2^, ultimately leading to performance degradation of the fused silica surface [18,19]. Raman et al. [20] investigated the mechanism of laser-induced damage produced by metal particles on fused silica surfaces, which can cause the formation of ablation pits on the surface, leading to the generation of a subsequent three-fold light intensity modulation, and the combined action ultimately causes damage. To elucidate the governing interaction mechanisms as a function of material and laser energy, time-resolved imaging was used to capture the particle ejection and expansion of the particles on the fused silica surface, the modification of the fused silica surface was studied due to exposure to the plasma generated by the particles and subsequent deposition of laser energy in the plasma. The laser energy deposition on the particle affects the energy redistribution and the modifications to the fused silica surface [21].

If this type of contamination control is performed, the source of metal contamination on the surface of the optical element must be identified. In the NIF device performance evaluation report, it was highlighted that particulate contaminants in the amplifier were the main cause of Nd glass damage. 

The main components of the particles are presumed with a scanning electron microscope (SEM) to be carbon particles, which are formed by the coalescence of carbon particles formed by the carbonization of the non-volatile residues (NVR) remaining on the surface of the mechanical components under the irradiation of a strong xenon lamp light [3]. Bude et al. elaborated on the phenomenon of particle contamination deposition within the final optical assembly and showed that contamination during the laser system operation can significantly reduce the damage threshold of the optical element [21]. Scattered light inevitably exists during the laser device operation, and aluminum alloys are commonly employed in laser facilities. When high-energy stray light irradiates the surface of aluminum alloys, a large amount of particle contamination ejects from the surface. If such particle contaminants settle freely on the surface of an optical element, they can damage the optical surface under laser irradiation [12]. The nanosecond laser ablation of metal surfaces has also been reported. When laser energy is used on the surface of a metallic material, the material is rapidly heated and melted, and a superheated state is formed; phase explosion ablation occurs near the material surface and detaches from the surface [12,21,22,23,24]. The mechanism of particle contaminant generation from aluminum alloy materials at critical laser energies has not yet been studied. Therefore, it is necessary to understand this mechanism, develop stray light control initiatives, and control the cleanliness of high-power laser facilities.

In this study, the morphologies of aluminum alloy materials irradiated with 355 and 1064 nm incident lasers were studied. The morphologies were characterized using optical microscopy. A profilometer and a field-emission SEM with an energy dispersive spectrometer (EDS) were used to obtain three-dimensional morphological details of the damage sites and components of the surface. In addition, we investigated the relationship between the number of particles produced on the surfaces of aluminum alloy materials with different laser energies and roughness values. Finally, numerical calculations of particle adsorption and laser cleaning on aluminum alloy surfaces can explain the observed experimental phenomena. The results show that appropriate aluminum alloy surface roughness and laser cleaning methods can prevent particle contamination caused by scattered light inside the laser facility.

## 2. Experiment

### 2.1. Sample Preparation

Aluminum alloy samples (as shown in Table 1) with dimensions of 50 × 50 mm and a thickness of 3 mm were prepared using the milling machining method, and the surface roughness values were 1.6, 3.2, and 6.4 μm. The surfaces of aluminum alloy samples were cleaned using 17 MPa ultrapure water combined with Brulin1990GD cleaning solvent for 10 min to remove particle contaminants with surface sizes greater than 50 microns [25]. The ultrapure water should have a conductivity of 15 MΩ. Subsequently, 28, 40, 108, and 120 Khz compound ultrasonic cleaners combined with Brulin815 GD solvent (Brulin, Indianapolis, IN, USA) were used to remove surface traces of organic and particle contaminants larger than one micron in size. Finally, the surface moisture was removed by baking using an infrared lamp at 200 °C for approximately 2 h [26]. A surface-cleanliness test was performed, and the sample surface was required to reach a cleanliness level of 100-A/10 to exceed the operational requirements of the laser facility [26].

### 2.2. Laser Damage Experiments

**Figure 1:**
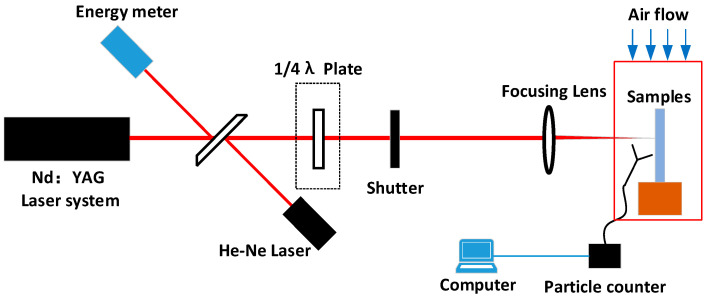
Schematic of laser-induced sputtering particle testing on aluminum alloy surface.

Laser damage experiments were conducted using an Nd:YAG laser. A Gaussian laser beam of 5 ns (1/e^2^) at wavelengths of 355 and 1064 nm was irradiated onto the surface of the aluminum alloy sample, and the spatial beam had a Gaussian distribution with a spot size of 1 mm^2^. The aluminum alloy sample to be tested was placed inside a small laminar flow hood, and a laser particle counter probe was placed under the hood, as shown in Figure 1. The velocity of clean air at the top of the laminar flow hood is approximately 0.5 m/s, and the static cleanliness class is better than ISO class 1, ensuring that the particle contaminants generated by the aluminum alloy under laser irradiation can be tested by the laser particle counter in its entirety, and no new contamination is introduced.

The “s-on-1” based laser damage test method was performed to determine the number of particles generated on the aluminum alloy surface. For each irradiation round, laser dust particle counting was performed to determine the particle contaminants produced by the laser irradiation. When the laser particle counter showed no particles, the next round of equal-flux irradiation was performed. The laser energy was monitored in real-time during the test using an energy calorimeter, and the laser energy was mediated with a quarter-wave plate.

### 2.3. Microscopic Morphological Characterization

The surface microstructures of the aluminum alloys before and after laser irradiation were observed using an optical microscope. To obtain a detailed morphology after multiple irradiations, we used a profilometer and scanning electron microscopy (5 to 18 kV) for analytical tests. The elemental composition of the aluminum alloy surface was established via the SEM-EDS analysis.

## 3. Experimental Results

### 3.1. Laser Damage Morphology Microstructure 

**Figure 2:**
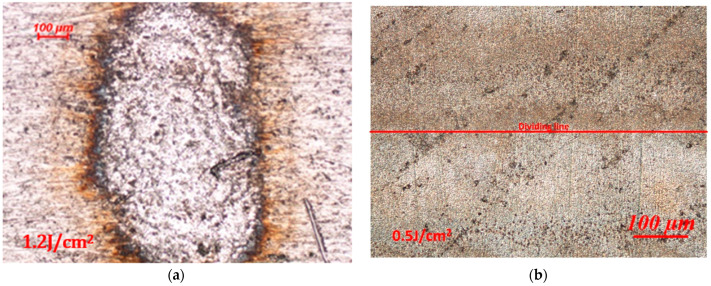
Optical micrographs of aluminum alloy under laser irradiation: (**a**) high-energy laser-irradiated area and (**b**) low-energy laser-irradiated area.

The microscopic morphology of the aluminum alloy surface after laser irradiation is shown in Figure 2. The damage morphologies differed due to the laser energy. In Figure 2a, thermal ablation occurred on the surface of the aluminum alloy under irradiation of a high-energy laser. Under lower laser energy, the thermal ablation effect was less significant, and there was no obvious ablation trace, as shown in Figure 2b [27,28]. However, a clear demarcation line for the laser irradiation could be observed under a microscope. The color of the laser-irradiated area darkened under the microscope, whereas it was obviously brighter in the unirradiated area. The mechanism of this phenomenon is closely related to the changes in the microstructure after laser irradiation.

### 3.2. Particle Contamination from Laser Irradiation

**Figure 3:**
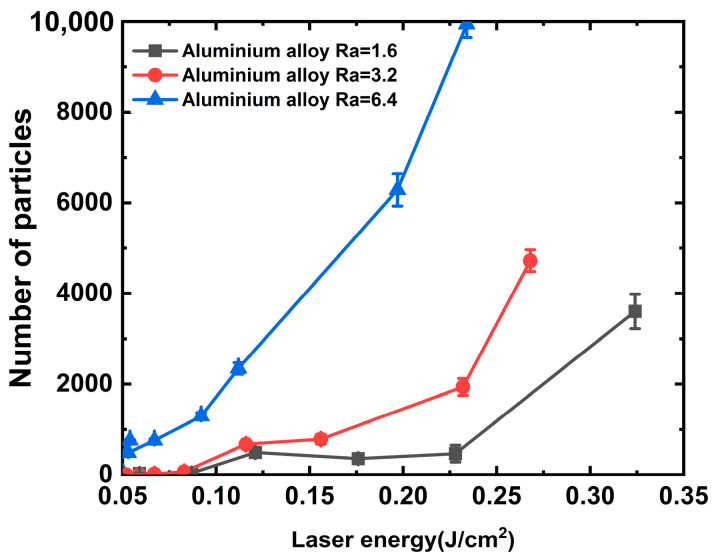
Relationship between particle contaminants and 1064 nm laser energy.

**Figure 4:**
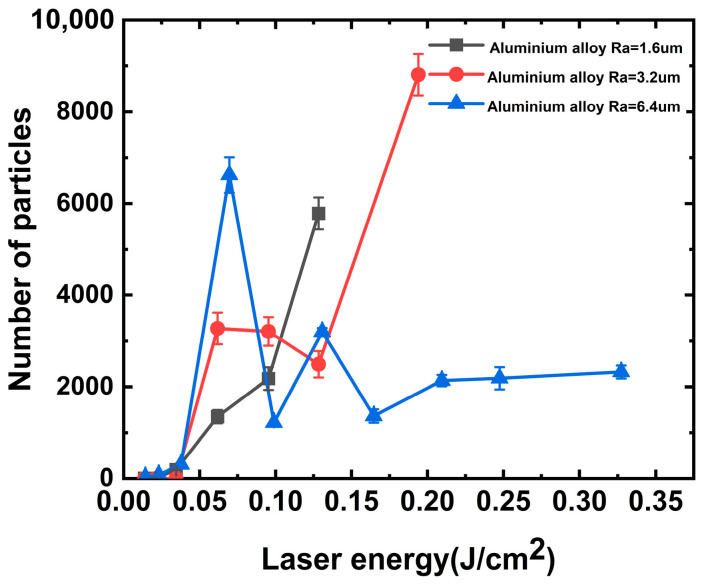
Relationship between particle contaminants and 351 nm laser energy.

To better understand the mechanism of particle generation via laser association with aluminum alloys, aluminum alloys with different roughness values (Ra of 1.6, 3.2, and 6.4) were irradiated with a laser at wavelengths of 1064 and 351 nm, and the number of particles generated (0.5 um particles for example [29]) were completely counted using a laser particle counter, and the results are shown in Figure 3 and Figure 4. It can be observed from the figures that the laser threshold of particles generated from the aluminum alloy under the action of the 1064 nm laser decreases with the improvement in roughness. Under a roughness of 1.6 μm, the initial particle generation threshold of the aluminum alloy is approximately 0.121 J/cm^2^ and generates 491 particles, as shown in Figure 3. However, under a roughness of 3.2 μm, the laser energy of the initial particle generation of the aluminum alloy is approximately 0.116 J/cm^2^ and generates 673 particles. We argue that the variation in the particle number due to the roughness is approximately 37% under the same laser energy irradiation. However, the initial laser energy for particle generation of the aluminum alloy at 6.4 μm roughness is approximately 0.054 J/cm^2^, and a significant quantity of particles can be generated at this energy. A single laser irradiation can generate 755 particles, and the threshold value for particle generation is approximately 0.05 J/cm^2^. Furthermore, the number of particles and laser energy exhibited an exponential growth relationship. In addition, we similarly observed that when the roughness is below 3.2 μm and the laser energy is lower than 0.225 J/cm^2^, a phenomenon occurred where particles produced using aluminum alloy and the irradiation laser energy increase first and then decrease, which is associated with the mechanism of the laser cleaning threshold of particles.

However, the aluminum alloy generated particulate contaminants when irradiated with the 351 nm laser, as shown in Figure 4. In this case, the laser energy required for particle generation in the aluminum alloy was approximately 0.065 J/cm^2^. Consistent with the previous discussion, the greater the roughness, the higher the number of particles generated. Under such laser threshold irradiation, the surface of aluminum alloy with a roughness of 6.4 nm produced approximately 4.90 and 2.02 times more particles under single-shot laser irradiation than that with a roughness of 1.6 and 3.2 μm. With increasing laser energy, we observed that the number of particles generated at laser energy irradiation values between 0.1 and 0.12 J/cm^2^ was relatively lower. We assumed that the particle contaminants embedded in the surface were removed from the aluminum alloy surface under strong laser irradiation. Simultaneously, under this laser energy irradiation, it is not sufficient to cause damage to the aluminum alloy surface to produce a large number of new particles, so there appears to be a zone of the energy plateau. 

**Figure 5:**
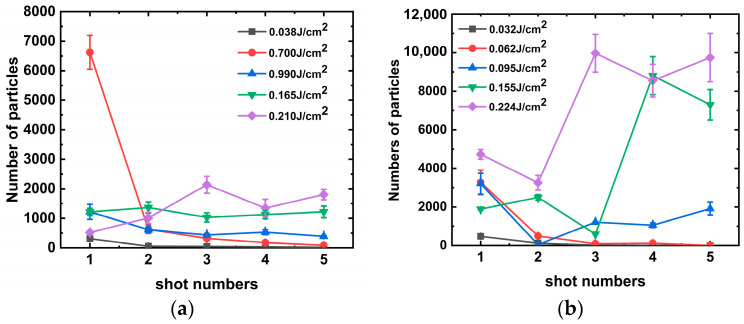
Number of particles produced by single-shot laser irradiation of aluminum alloy at 351 nm: (**a**) Ra = 6.4 and (**b**) Ra = 3.2.

The stray light within the final optical assembly is primarily a laser with a wavelength of 351 nm [30]; thus, we focused on the mechanism of particle generation by multiple irradiations of the aluminum alloy with this wavelength, and the results are shown in Figure 5. The particles of aluminum alloys with roughness values of 6.4 and 3.2 μm were tested using the “s-on-1” method at different laser energy irradiations. When the laser irradiated the aluminum alloy with a roughness of 6.4 um on a single point with multiple shots, the number of particles produced decreased gradually with the increase in irradiation shots because the surface embedded particle contaminants were cleaned with the laser. It is assumed that laser cleaning plays a dominant role in this process.

However, when the irradiation laser energy was increased to 0.165 J/cm^2^, the number of particles produced by each laser irradiation was approximately the same. It can be assumed that laser-induced surface damage of the aluminum alloy occurred at this laser energy. As the laser energy continued to increase, damage occurred during the third laser irradiation, resulting in the production of a larger amount of particulate matter.

Similarly, damage to the aluminum alloy occurs between the third and fourth shots under 351 nm laser irradiation. Damage occurs at a further reduced energy of approximately 0.095 J/cm^2^ at a roughness of 3.2 μm. We consider the possibility that spike-like structures formed on smooth surfaces with low roughness are more susceptible to damage and that the number of particles produced by a single laser irradiation is approximately 8000.

## 4. Discussion

### 4.1. Particle Spattering Mechanism under Strong Laser Interaction

**Figure 6:**
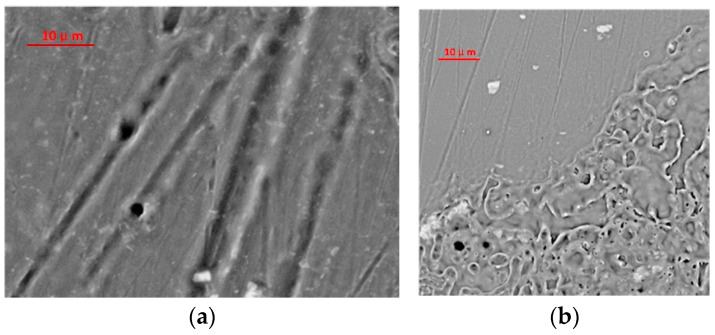
Three-dimensional morphology of aluminum alloy surface under the action of different laser energies: (**a**) 0.05 J/cm^2^ laser irradiation SEM image and (**b**) 0.2 J/cm^2^ laser irradiation SEM image.

**Figure 7:**
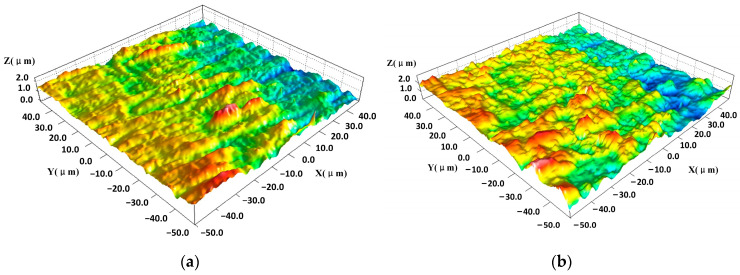
Three-dimensional morphology of aluminum alloy surface: (**a**) initial and (**b**) after laser irradiation about 0.05 J/cm^2^.

Figure 6 shows the three-dimensional microscopic morphology of the aluminum alloy under laser irradiation at different energies. In Figure 6a, the scanning electron microscope (SEM) image shows that multiple tip structures coexisted in the laser action area. Many grain lines on the surface of the aluminum alloy were formed during processing, and the particle contaminants generated during processing are interspersed within the grain lines, forming a subsurface-embedded particle contamination layer [31]. Thus, the surface is relatively flat, as shown in Figure 7a. The height of the surface protrusions is about 2 um. However, the particle contaminants in the grain lines have a strong absorption effect on the laser light; it absorbs laser energy and then breaks away from the aluminum alloy surface, forming particle spattering. When this debris detaches from the surface of the aluminum alloy, many spike-like structures are formed on the surfaces of the aluminum alloys, as shown in Figure 7b.

**Figure 8:**
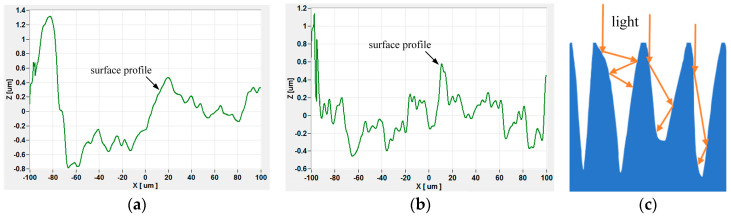
Two-dimensional morphology of aluminum alloy surfaces under laser irradiation and the reflection of light from the laser action area: (**a**) initial, (**b**) after laser irradiation about 0.05 J/cm^2^, and (**c**) the reflection of light with multi-spike structures.

The two-dimensional morphology of aluminum alloy surfaces before and after laser irradiation is shown in Figure 8a,b. The difference between the highest and lowest points on the surface is about 2.2 μm before the laser irradiation, but it is about 1.6 μm under the laser irradiation. And also, one can find many spike-like structures, which is the same in Figure 7b. Embedded particles formed between spikes during processing were completely removed under laser irradiation, resulting in the exposure of the spike structure. Some of the spikes were also ablated and removed under laser irradiation, forming a trapped light structure, as Figure 8c shows.

**Figure 9:**
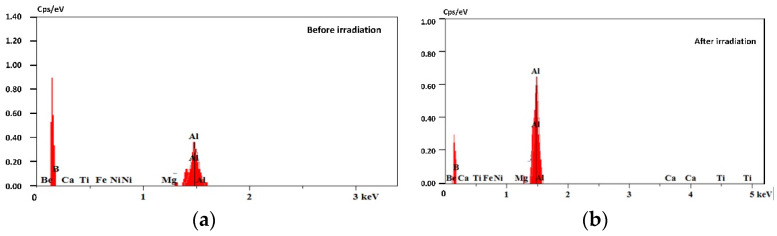
EDS spectra of the aluminum alloy surfaces: (**a**) initial and (**b**) after laser irradiation.

By comparing with Figure 2b, the laser-irradiated part shows a distinct dull color under bright-field microscope observation, indicating a lower reflectivity in this region, whereas the non-irradiated area has a distinctly higher reflectivity. Its minus-reflection effect is shown in Figure 8c. Such structures have the effect of beam traps when light irradiates the surfaces of spike-like structures [32]. When the laser energy reaches a certain level of approximately 0.2 J/cm^2^, the machined surface of the aluminum alloy is heated and melted with the laser, as shown in Figure 6b. EDS analysis showed an elemental composition of the aluminum alloy, as presented in Table 1 and the spectra of Figure 9a,b. The elemental compositions of the surface were found to be 99.16% aluminum and 0.84% magnesium. However, after laser irradiation, the elemental compositions of the surface were found to be 98.52% aluminum and 1.48% magnesium. The detected elements were evidenced with the EDS spectra presented in Figure 9. The spectra show that the compositions of the aluminum alloy surface did not change significantly during laser irradiation. We assume that the aluminum alloy was removed greatly during laser irradiation, or the elemental compositions of the embedded particles are aluminum. 

### 4.2. Theoretical Model

The surface cleanliness after precision cleaning can reach the level of BJD 100-A/10. The spectrum shows that no other components were found on the surface, as Figure 9a shows. Therefore, the particles are assumed to be aluminum alloys in the theoretical model. The adsorption force of the particles within the metal subsurface processing layer can be approximated as the van der Waals force. According to the adsorption and deformation mechanism [33], the adsorption force on the particles is determined by considering the deformation variables, which can be expressed as follows:

(1) $$F_{ad}=F_{o}+F_{d}=\frac{Ar}{6H^{2}}\left(1+\frac{r_{c}^{2}}{rH}\right)$$

where *F*_0_ and *F_d_* are the van der Waals forces without and caused by deformation, respectively. a is the system Hamaker constant, r represents the particle diameter, *H* represents the distance between the particle and surface (typically taken as 0.4 nm), and *r_c_* represents the contact radius between the particle and substrate surface caused by deformation. Based on the Derjaguin theory, the relationship between the particle radius and contact radius is as follows:

(2) $$r_{c}^{3}=\left(\frac{Ar^{2}}{8E^{*}H^{2}}\right)$$

(3) $$\frac{1}{E^{*}}=\left(\frac{1-\sigma_{1}^{2}}{E_{1}}+\frac{1-\sigma_{2}^{2}}{E_{2}}\right)$$

where *σ*_1,2_ and *E*_1,2_ represent the Poisson coefficient and Young’s modulus, respectively, and Table 1 and Table 2 identify the particle and substrate, respectively. Subsequently, the ratio of the contact radius to the particle radius is as follows:

(4) $${\left(\frac{r_{c}}{r}\right)}^{3}=\left(\frac{A}{8E^{*}H^{2}r}\right)$$

According to Equations (1)–(4), the contact radius for particle adsorption is considered to be 2 *r_c_*; therefore, the adsorption force of the particles on the aluminum alloy surface can be calculated.

The temperature increase caused when the laser beam irradiates the aluminum alloy surface can be described by a simplified one-dimensional heat conduction Equation [34], as shown in Equation (5), where the temperature distribution *T*(*x,t*) is a function of the depth x and irradiation time t as follows:

(5) $$\rho c\frac{\partial T(x,t)}{\partial t}=k\frac{\partial^{2}T(x,t)}{\partial x^{2}}+(1-R)\alpha I_{0}\exp(-\alpha x)$$

where *ρ*, *c*, *k*, *R*, and *αI*_0_ represent the density, specific heat, thermal conductivity, reflectivity, and absorption coefficient, as shown in Table 2.

Because the reflectivity of aluminum alloys with different laser roughness differs, the reflectivity of the aluminum alloy was tested using a spectrophotometer, and the test results are shown in Table 3.

**Figure 10:**
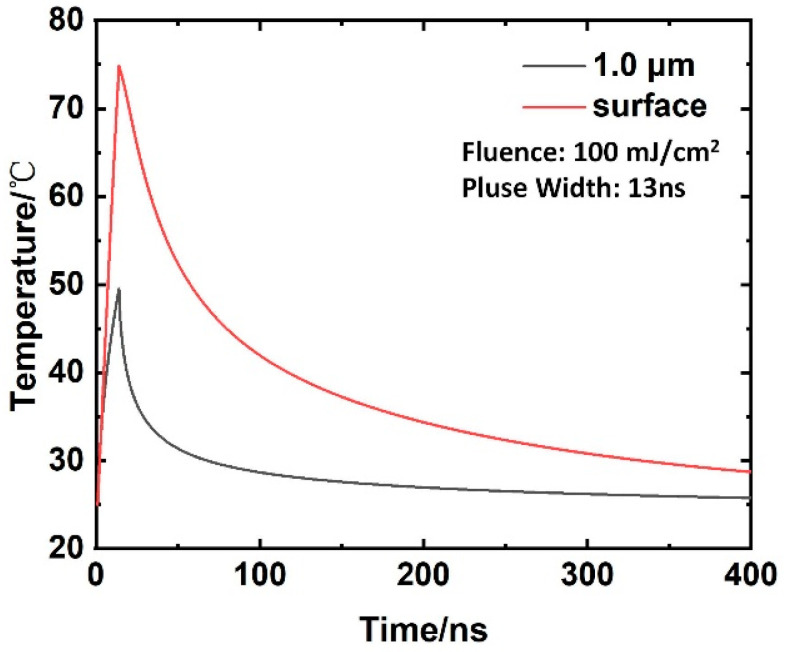
Temperature distribution in Al substrates for different depths and times at 100 mJ/cm^2^.

According to the parameters of Equation (5), Table 1 and Table 3, the temperature field distribution of the aluminum alloy surface under laser irradiation can be calculated and obtained, as shown in Figure 10. From Figure 10, it can be observed that the temperature reaches the apex at the same time as the pulse width, and the highest point temperature of the surface is approximately 75 °C. When the depth is 1 um below the surface, the maximum temperature is about 50 °C. The surface temperature of the aluminum alloy returned to room temperature within approximately 300 ns of laser irradiation, and the recovery time was much shorter than the subsequent pulse irradiation time interval.

**Figure 11:**
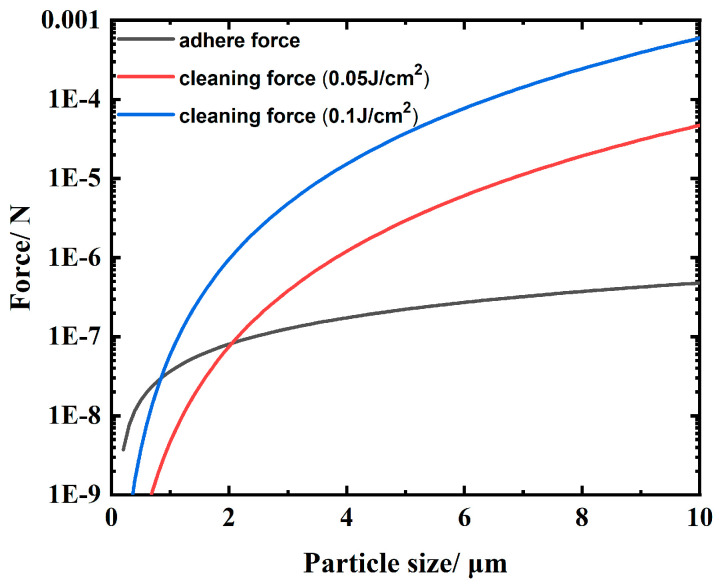
Cleaning force induced by laser irradiation and the adsorption force for different particle diameters.

The thermal expansion scale *H* of the particles can be calculated based on the temperature rise of the particles as follows:

(6) H=aμΔT

where *a* and *μ* represent the thermal diffusion coefficient and length during laser pulse irradiation, respectively. The thermal diffusion coefficient is a = k/ρc. However, if this expansion is achieved with a laser pulse duration of 13 ns, so are the average velocity and acceleration, respectively. The cleaning force on the particle surface can be approximated with the velocity and acceleration [35], as shown in Figure 11. Under the action of laser energy of 0.05 J/cm^2^, the particle contaminants embedded in the aluminum alloy surface with a scale larger than 2 μm can be effectively removed. When the laser energy is raised to 0.1 J/cm^2^, the scale of cleaned particle contaminants can reach up to about 1 μm, which is basically consistent with the experimental observation.

When the particles embedded in the aluminum alloy surface are completely cleaned, the surface is exposed to a machined groove-type structure, as shown in Figure 8. The absorption of this structure by the subsequent laser irradiation increased sharply to approximately 100%. According to Equation (5) and the description of references [13,28,36], the temperature rises on the surface of aluminum alloy material can reach 796.22 °C under the action of 0.2 J/cm^2^ laser energy, which exceeds the melting point of aluminum material 660 °C. This can better explain the experimental phenomenon in which the threshold value of a large amount of particle contaminants generated on the surface of the aluminum alloy in Figure 3, Figure 4 and Figure 5 is near 0.2 J/cm^2^.

## 5. Conclusions

The machining material embedded in the surface of the aluminum alloy is ejected by stray light irradiation, which is the primary reason for dynamic particle contamination inside a high-power laser. Scanning electron microscope test results have confirmed that under the influence of laser energy of 0.1 J/cm^2^, the particles embedded inside the processing traces are removed under the action of laser cleaning, and subsequently, the absorption of laser light on the material surface increases sharply. The damage threshold of the material surface is approximately 0.2 J/cm^2^, and the particle contaminant generated per unit area after damage is approximately 1000 particles/mm^2^. When the laser energy reaches higher intensities, the ablation effect on the aluminum alloy surface is obvious with each irradiation, forming a crater and resulting in significant amounts of sputtering debris. Three-dimensional morphology of aluminum alloy surfaces before and after laser irradiation disclose the source of the spattering debris, which was embedded in the subsurface during processing. When debris embedded in the subsurface of the aluminum alloy is removed, light-trapped microstructures are formed on the surface. When subsequent laser irradiation of the equivalent energy irradiates the surface of the aluminum alloy, the probability of ablation increases dramatically. EDS elemental analysis results demonstrated the identical spectrum on the surface of the aluminum after laser irradiation with no compositional variation. The relationships among the laser cleaning force, adsorption force, and particle size were numerically calculated to determine the primary mechanism of particle spattering on the material surface. This study can be used to control aerosol concentrations in high-power laser facilities, extend the life of internal optics, and reduce the operating costs of the facilities. And there is provided an approach for removing embedded particles from the subsurface of aluminum alloy.

## Figures and Tables

**Table 1:** Nominal chemical composition of 5083 Al alloy (wt%).

Alloy	Al	Si	Cu	Mg	Zn	Mn	Ti	Cr	Fe
5083	Bal.	0.40	0.10	4.0–4.9	0.25	0.4–1.0	0.15	0.05–0.25	0.4

**Table 2:** Material parameters for calculation.

Parameter	Value
Density/(kg/m^3^)	2770
Specific heat/(J/kg·K)	875
Thermal conductivity/(W/m·K)	190
Poisson coefficient	0.33
Young’s modulus/Gpa	70.3
Hamke constant(Al-Al)	33 × 10^−20^
Thermal expansion coefficient/K	23.21 × 10^−6^

**Table 3:** Reflectance of different roughness.

Roughness	Reflectance at 1064 nm	Reflectance at 351 nm
1.6 μm	23.64	12.85
3.2 μm	26.97	16.64

## Data Availability

The datasets generated during and/or analyzed during the current study are available from the corresponding author on reasonable request.
